# A novel family of lifetime distribution with applications to real and simulated data

**DOI:** 10.1371/journal.pone.0238746

**Published:** 2020-10-01

**Authors:** Muhammad Ijaz, Wali Khan Mashwani, Samir Brahim Belhaouari

**Affiliations:** 1 Department of Statistics, University of Peshawar, Peshawar, Pakistan; 2 Institute of Numerical Sciences, Kohat University of Science &Technology, Kohat, Pakistan; 3 Division of Information and Computing Technology, College of Science and Engineering, Hamad Bin Khalifa University, Ar-Rayyan, Qatar; Tongii University, CHINA

## Abstract

The paper investigates a new scheme for generating lifetime probability distributions. The scheme is called Exponential- H family of distribution. The paper presents an application of this family by using the Weibull distribution, the new distribution is then called New Flexible Exponential distribution or in short NFE. Various statistical properties are derived, such as quantile function, order statistics, moments, etc. Two real-life data sets and a simulation study have been performed so that to assure the flexibility of the proposed model. It has been declared that the proposed distribution offers nice results than Exponential, Weibull Exponential, and Exponentiated Exponential distribution.

## Introduction

Probability distribution plays a vital role in modeling lifetime data that arise in different fields of science such as in Survival analysis, Economics, Biology, Engineering, and in some other applied field of sciences. There are many lifetime probability distributions that can be used to model the data, for example, Exponential, Weibull, and Weibull Exponential distribution are among others. All these distributions have desirable properties and real applications. However, these distributions fail to model the data following a non-monotonic hazard rate function, for example, Exponential distribution can only model the constant hazard rate and the Weibull distribution can only model a monotonic hazard rate function. In this paper, we have present a new distribution that can model both the monotonically and non-monotonically hazard rate functions. But in practice, we have real data sets which follow a non-monotonic hazard rate function, for example, the infant mortality rate, or the lifetime of an electronic device follows a non-monotonic hazard rate functions.

To overcome the above limitations found in the existing probability distributions, researchers are working to modify these distributions. It is usual practice to modify the current distributions by generating a generator and then applied to the existing distributions so as to derive a new probability model. For example, Aldeni et. al [1] produced a new family of distributions arising from the quantile of generalized lambda distribution, Cordeiro et. al [2] worked on the generalized odd half-Cauchy family of distributions, Alzaatreh et. al [3] presented a generalized Cauchy family of distributions, Alzaatreh et. al [4] introduced T-normal family of distributions, Nasir et. al [5] investigated the generalized Burr family of distributions based on quantile function, Mudholkar et. al [6] worked on the Exponentiated Weibull family of distribution.

This paper contributes a new scheme for generating probability distributions to the existing literature of probability theory. In this paper, a new scheme is investigated and applied to the existing probability distributions so as to derive a new probability distribution. The main objective of the paper is to achieve maximum flexibility while modeling the lifetime data with both the monotonically and non-monotonically hazard rate functions.

Let *X* be a continuous random variable follows the Exponential distribution then the cumulative distribution function (Cdf) is given by

F(x)=1−exp(−ax),x>0,a>0 (1.1)

The Exponential distribution is modified by many researchers, for example, Gupta and Kundu [7] presented the generalized distribution which is also known as Exponentiated Exponential distribution. The Cdf is given by

$$F\left(x\right)={\left(1-\exp\left(-\lambda x\right)\right)}^{\alpha },\;x,\lambda ,\alpha >0$$ (1.2)

Barreto, and Cribari [8] introduced a generalization of the Exponential-Poisson distribution with the following Cdf

$$F\left(x\right)=\frac{\left(1-\exp\left(-\lambda +\lambda \exp\left(-\beta x\right)\right)\right)}{1-\exp\left(-\lambda \right)},\;x,\lambda ,\beta >0$$ (1.3)

Barreto et. al [9] introduced the Beta Generalized Exponential distribution. El-Bassiouny [10] introduced the Exponential Lomax distribution. Mudholkar and Srivastava, (1993) defined the Exponentiated Weibull family of distribution [6]. Nadarajah and Kotz [11] present the Beta Exponential distribution.

In this paper, a novel family is produced called Exponential- H family of distribution. We discussed one special case of this family and call it New Flexible Exponential distribution (NFE) by employing the Weibull distribution as a baseline. The detailed discussion is as follows

## Exponential- H family (Ex-H) of distributions

The Exponential- H family (Ex-H) is mostly related to the Weibull-G family of distributions investigated by Marcelo et. al [12]. The cumulative distribution function (CDF) of the Exponential- H family (Ex-H) takes the following form

G(x,a,ζ)=1−exp(−aL(x;ζ)),x,a>0 (2.1)

where *L*(*x*;*ζ*) = *H*(*x*;*ζ*)*exp*(*x*), and *H*(*x*;*ζ*) is the non-decreasing function hazard rate function depending on the parameter vector *ζ*. The corresponding probability density function (PDF) is given by

g(x,a,ζ)=aexp(−aL(x;ζ))l(x;ζ),x,a>0 (2.2)

## New Flexible Exponential distribution (NFE)

This section illustrates the special case of the Ex-H family by considering the hazard function of the Weibull distribution. The hazard function of the Weibull distribution is defined by

$$H\left(x;\zeta \right)=ax^{b-1}$$

By employing the above result in Eq (2.1) and (2.2), we obtained the CDF and PDF of the NFE distribution respectively

$$F\left(x,a,b\right)=1-exp\left(-a^{2}bx^{\left(b-1\right)}exp\left(x\right)\right),\;x>0,b>1,a>0$$ (3.1)

$$f\left(x\right)=a^{2}bx^{\left(b-2\right)}\left(x+b-1\right)exp\left(x-a^{2}bx^{b-1}exp\left(x\right)\right),\;x>0$$ (3.2)

The survival and hazard rate function of NFE is defined by

$$S\left(x\right)=exp\left(-a^{2}bx^{b-1}exp\left(x\right)\right)$$ (3.3)

$$h\left(x\right)=a^{2}bx^{b-2}\left(x+b-1\right)exp\left(x\right)$$ (3.4)

**Fig 1:**
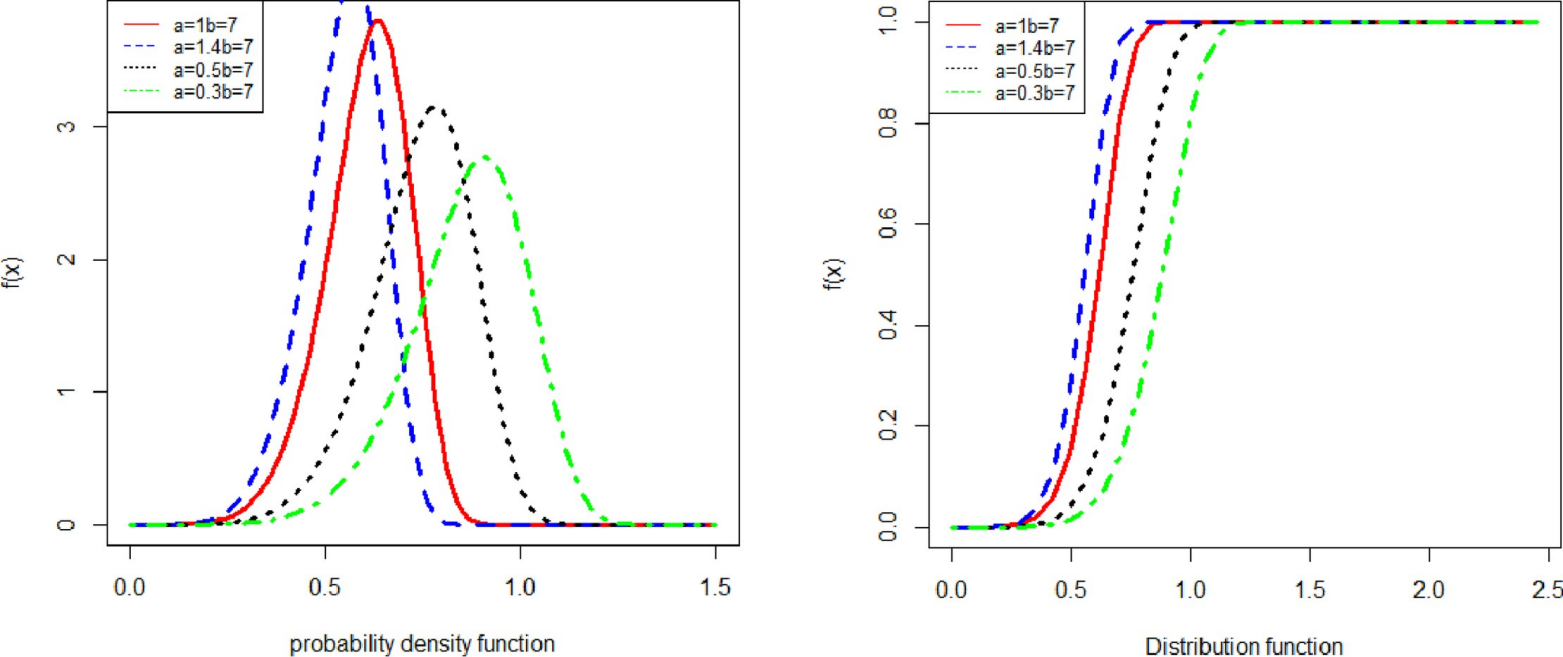
The Pdf and Cdf of NFE.

Fig 1 shows the graphical representation of the probability density function and cumulative distribution function, with different parameter values.

**Theorem 1.** The behavior of the hazard rate *h*(*x*) function of NFE (*a*,*b*) is defined by

- Increasing when *a*>0,*b*>1,
- Decreasing when *a*>0,*b*≤1.

Proof. The derivative of Eq (3.4) is given by

$$h^{\prime }\left(x\right)=a^{2}bx^{b-2}\left(b^{2}+b\left(2x-3\right)+x^{2}-2x+2\right)exp\left(x\right)$$

For *a*>0,*b*≤1,*h*′(*x*). Then the function *h*(*x*) is decreasing and for *a*>0,*b*>1. For *a*>0,*b*>1, *h*′(*x*) = 0 implies that the *h*(*x*) has a maximum at

$$x=1-b\pm \sqrt{b-1}$$

and, the function *h*(*x*) is increasing for *a*>0,*b*>1,

Hence, the hazard rate function has the ability to model both monotonically and non-monotonically hazard rate functions.

**Fig 2:**
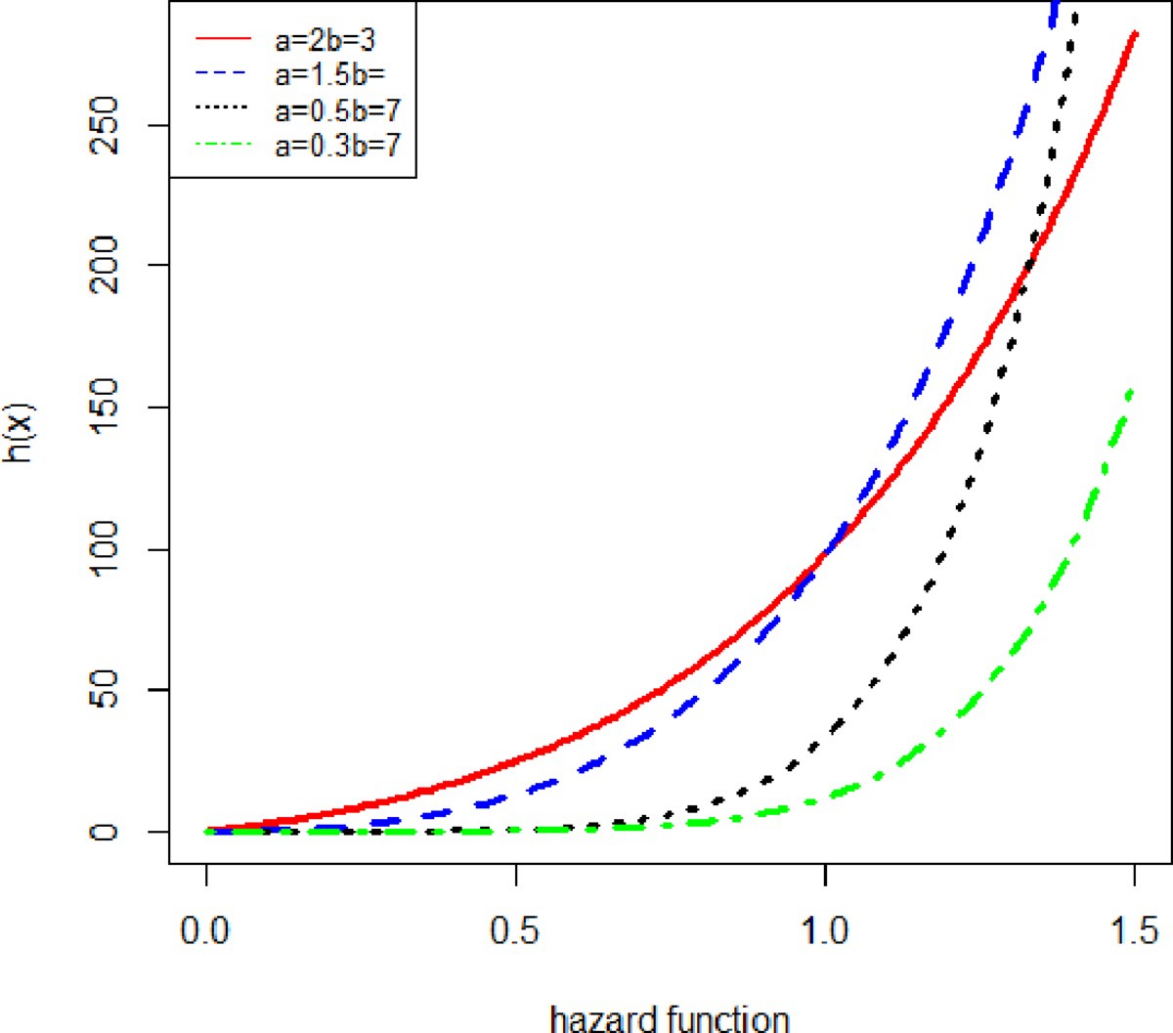
Hazard function of NFE.

Fig 2 shows the plot for the hazard function of the New Flexible Exponential distribution with different values of a parameter.

## Quantile function and median

The quantile function *Q*_(*NFE*)_(*x*) of the *NFE*(*a*,*b*) is the real solution of the following equation

F(x)=u

$$1-exp\left(-a^{2}bx^{b-1}exp\left(x\right)\right)=u$$ (4.1)

where *u*~Uniform (0,1).

Solving (4.1) for *x*, we have

$$x=\left(b-1\right)W\left(\frac{{\left(\frac{-\log\left(1-u\right)}{a^{2}b}\right)}^{1/\left(b-1\right)}}{b-1}\right)$$ (4.2)

where W (Z) is the Lambert W function and is defined as

$$W\left(z\right)=\frac{\sum_{n=1}^{\infty }{\left(-1\right)}^{n}n^{n-2}}{\left(n-1\right)!}z^{n}.$$

For the median, put *u* = 0.5 in Eq (4.2).

## R^th^ moments

**Theorem 2**: If a random variable *X* has NFE distribution with parameters *a*,*b* then the r^th^ moments (about the origin) of *X* is defined by

$$u_{r}^{\prime }=\sum_{k=0}^{\infty }\frac{{\left(‐1\right)}^{k}}{k!}{\left(a^{2}b\right)}^{k+1}\left[\frac{\Gamma \left(r+b+bk‐k\right)}{{\left(k+1\right)}^{r+b+bk‐k}}+\left(b-1\right)\frac{\Gamma \left(r+b+bk-k-1\right)}{{\left(k+1\right)}^{r+b+bk-k-1}}\right]$$

Proof. We know that

$$u_{r}^{\prime }=E(x^{r})=\int_{0}^{\infty }x^{r}f(x)dx$$

Putting (3.2) in the above expression, we obtained the following form

$$u_{r}^{\prime }=\int_{0}^{\infty }\left(x^{r}a^{2}bx^{\left(b-2\right)}\left(x+b-1\right)exp\left(x-a^{2}bx^{b-1}exp\left(x\right)\right)\right)dx$$

$$\begin{array}{l} =\int_{0}^{\infty }\left(x^{r+b-1}a^{2}bexp\left(x-a^{2}bx^{b-1}exp\left(x\right)\right)\right)dx+b-1\int_{0}^{\infty }\left(x^{r+b-2}a^{2}bexp\left(x-a^{2}bx^{b-1}exp\left(x\right)\right)\right)dx \end{array}$$ (5.1)

Solving the first part in the above expression (5.1), we have

$$=\int_{0}^{\infty }\left(x^{r+b-1}a^{2}bexp\left(x-a^{2}bx^{b-1}exp\left(x\right)\right)\right)dx$$

$$=\int_{0}^{\infty }\left(x^{r+b-1}a^{2}bexp\left(x\right)exp\left(-a^{2}bx^{b-1}exp\left(x\right)\right)\right)dx$$

$$=\sum_{k=0}^{\infty }\frac{{\left(-1\right)}^{k}}{k!}{\left(a^{2}b\right)}^{k+1}\int_{0}^{\infty }\left(x^{r+b+bk+k-1}exp\left(\left(k+1\right)x\right)\right)dx$$

$$=\sum_{k=0}^{\infty }\frac{{\left(‐1\right)}^{k}}{k!}{\left(a^{2}b\right)}^{k+1}\frac{\Gamma \left(r+b+bk‐k\right)}{{\left(k+1\right)}^{r+b+bk‐k}}$$ (5.2)

Now solving the second part of (5.1), we have

$$=b-1\int_{0}^{\infty }\left(x^{r+b-2}a^{2}bexp\left(x-a^{2}bx^{b-1}exp\left(x\right)\right)\right)dx$$

$$=\sum_{k=0}^{\infty }\frac{{\left(‐1\right)}^{k}}{k!}{\left(a^{2}b\right)}^{k+1}\left(b-1\right)\frac{\Gamma \left(r+b+bk-k-1\right)}{{\left(k+1\right)}^{r+b+bk-k-1}}$$ (5.3)

By combining (5.2) and (5.3) the result has obtained

$$u_{r}^{\prime }=\sum_{k=0}^{\infty }\frac{{\left(‐1\right)}^{k}}{k!}{\left(a^{2}b\right)}^{k+1}\left[\frac{\Gamma \left(r+b+bk-k\right)}{{\left(k+1\right)}^{r+b+bk-k}}+\left(b-1\right)\frac{\Gamma \left(r+b+bk-k-1\right)}{{\left(k+1\right)}^{r+b+bk-k-1}}\right]$$

## Order statistics

Let *X*_1_,*X*_2_,*X*_3_,…*X*_*n*_ be ordered random variables, then the Pdf of the *i*^*th*^ order statistics is given by,

$$f_{\left(i;n)(x\right)}=\frac{n!}{(i-1)!(n-i)!}f(x)F{\left(x\right)}^{\left(i-1\right)}{\left[1-F(x)\right]}^{\left(n-i\right)},$$ (6.1)

The 1^st^ and n^th^ order probability density function of NFE can be obtained by putting (3.1) and (3.2) in (6.1), and is given by respectively

$$f_{\left(1:n\right)}(x)=n\left(a^{2}bx^{\left(b-1\right)}\left(x+b-1\right)exp\left(x-a^{2}bx^{b-1}exp\left(x\right)\right)\right){\left(exp\left(-a^{2}bx^{b-1}exp\left(x\right)\right)\right)}^{\left(n-1\right)}$$ (6.2)

$$f_{\left(n:n\right)}(x)=n\left(a^{2}bx^{\left(b-1\right)}\left(x+b-1\right)exp\left(x-a^{2}bx^{b-1}exp\left(x\right)\right)\right){\left(1-exp\left(-a^{2}bx^{b-1}exp\left(x\right)\right)\right)}^{n-1}$$ (6.3)

## Parameter estimation

In this section, the maximum likelihood method is used to find out the estimates of the unknown parameters of NEF (a, b) based on a complete data set information. Let us assume that we have a sample *X*_1_,*X*_2_,*X*_3_…*X*_*n*_ from NEF (*a*,*b*). The Likelihood function is given by

$$L=\prod_{i=1}^{n}f\left(x_{i};a,b\right),\;\mathrm{where}\;a,b>0$$ (7.1)

Substituting (3.2) in (7.1), we get

$$L=\prod_{i=1}^{n}\left(a^{2}bx^{\left(b-2\right)}\left(x+b-1\right)exp\left(x-a^{2}bx^{b-1}exp\left(x\right)\right)\right)$$ (7.2)

By applying the natural log to (7.2), the log-likelihood function is defined by

$$l=nlog\left(a^{2}b\right)+\left(b-2\right)\sum_{i=1}^{n}logx_{i}+\sum_{i=1}^{n}log\left(x_{i}+b-1\right)+\sum_{i=1}^{\infty }\left(x_{i}-a^{2}bx_{i}{}^{b-1}\exp(x_{i})\right)$$ (7.3)

To find the estimates of the unknown parameters, we have to compute the partial derivatives of (7.3) with respect to parameters and equate the results to zero

$$\frac{2n}{a}-2ab\sum_{i=1}^{n}\left(x_{i}^{b-1}exp\left(x_{i}\right)\right)=0$$ (7.4)

$$\frac{n}{b^{2}}+\sum_{i=1}^{n}\left(logx_{i}\right)+\sum_{i=1}^{n}\frac{1}{\left(x_{i}+b-1\right)}+\sum_{i=1}^{n}\left(-a^{2}x_{i}{}^{b-1}exp\left(x_{i}\right)\left(logx_{i}+1\right)\right)=0$$ (7.5)

The above two Eq (7.4) and (7.5) are not in closed form. Thus, it is difficult to estimate the unknown parameters and hence we refer to use the numerical technique that is the Newton Raphson or Bisection method to get the MLE.

## Asymptotic confidence bounds

Since, the MLE of the unknown parameters is not closed in form and thus the exact distribution of MLE cannot be derived. However, one can find the asymptotic confidence bounds for the unknown parameters of *NEF*(*a*,*b*) based on the asymptotic distribution of MLE which is as follows

The second time partial derivatives of Eq from (7.4) and (7.5) is respectively given by

$$\frac{\partial l}{\partial a^{2}}=I_{11}=-\frac{2n}{a^{2}}-2b\sum_{i=1}^{n}\left(x_{i}{}^{b-1}exp\left(x_{i}\right)\right)$$ (8.1)

$$\frac{\partial l}{\partial ab}=I_{12}=-2a\sum_{i=1}^{n}\left(x_{i}{}^{b-1}exp\left(x_{i}\right)\left(b\log x_{i}+1\right)\right)$$ (8.2)

$$\frac{\partial l}{\partial b^{2}}=I_{22}=-2\frac{n}{b^{3}}-\sum_{i=1}^{n}\left(\frac{1}{{\left(b+x_{i}-1\right)}^{2}}\right)+a^{2}\sum_{i=1}^{n}\left(x_{i}{}^{b-1}exp\left(x_{i}\right)\log x_{i}\right)\left(b\log x_{i}+2\right)$$ (8.3)

The observed information matrix is defined by

$$I=-\left(\begin{array}{cc} I_{11} & I_{12}\\ I_{21} & I_{22} \end{array}\right)$$

Hence, the variance-covariance matrix is approximated as

$$V=\left(\begin{array}{cc} v_{11} & v_{12}\\ v_{21} & v_{22} \end{array}\right)={\left(\begin{array}{cc} I_{11} & I_{12}\\ I_{21} & I_{22} \end{array}\right)}^{-1}$$

To obtain the estimate of V, we have to replace the parameters by the corresponding MLE, which is defined as

$$\overset{\wedge }{v}={\left(\begin{array}{cc} \overset{\wedge }{I_{11}} & \overset{\wedge }{I_{12}}\\ \overset{\wedge }{I_{21}} & \overset{\wedge }{I_{22}} \end{array}\right)}^{-1}$$ (8.4)

By using the above variance-covariance matrix, we can derive the (1 - β) 100% confidence intervals for the parameters *a* and *b* in the following form

$$\hat{a}\pm Z_{\frac{\beta }{2}}\sqrt{\mathrm{var}\left(\hat{a}\right)},\;\hat{b}\pm Z_{\frac{\beta }{2}}\sqrt{\mathrm{var}\left(\hat{b}\right)},$$

where $Z_{\frac{\beta }{2}}$ is the upper ${\left(\frac{\beta }{2}\right)}^{th}$ percentile of the standard normal distribution.

## Renyi entropy

**Theorem 3:** If a random variable *X* has *NFE*(*a*,*b*) then the Renyi entropy *R*_*H*_(*x*) is defined by

$$R_{H}\left(x\right)=\frac{1}{1-p}\log\left[{\left(a^{2}b\right)}^{p+j}\sum_{j=0}^{\infty }\sum_{k=0}^{\infty }\left(\begin{array}{c} p\\ k \end{array}\right)\frac{{\left(-1\right)}^{j}}{j!}{\left(b-1\right)}^{p-k}\right]\frac{\Gamma \left(p(b-2)+k+bj-j+1\right)}{{\left(-j-1\right)}^{\left(p(b-2)+k+bj-j+1\right)}}$$

Proof. The general form of the Renyi entropy is given by

$$R_{H}(x)=\frac{1}{1-p}\log\int_{0}^{\infty }f^{p}(x)dx$$

By employing (3.2) in the above expression, we have

$$=\frac{1}{1-p}\log{\int_{0}^{\infty }\left(a^{2}bx^{\left(b-2\right)}\left(x+b-1\right)exp\left(x-a^{2}bx^{b-1}exp\left(x\right)\right)\right)}^{p}dx$$

$$=\frac{1}{1-p}\log\left[{\left(a^{2}b\right)}^{p}{\int_{0}^{\infty }\left(x^{p\left(b-2\right)}{\left(x+b-1\right)}^{p}exp\left(x-a^{2}bx^{b-1}exp\left(x\right)\right)\right)}^{p}dx\right]$$ (9.1)

using the following Binomial and exponential expansion

$${\left(x+b-1\right)}^{p}=\sum_{k=0}^{\infty }\left(\begin{array}{c} p\\ k \end{array}\right)x^{k}{\left(b-1\right)}^{p-k}$$

and

$$exp{\left(x-a^{2}bx^{b-1}exp\left(x\right)\right)}^{p}=\sum_{j=0}^{\infty }\frac{{\left(-1\right)}^{j}}{j!}{\left(a^{2}bx_{i}{}^{b-1}exp\left(x_{i}\right)\right)}^{j}$$

After a few steps, we get

$$R_{H}\left(x\right)=\frac{1}{1-p}\log\left[{\left(a^{2}b\right)}^{p+j}\sum_{j=0}^{\infty }\sum_{k=0}^{\infty }\left(\begin{array}{c} p\\ k \end{array}\right)\frac{{\left(-1\right)}^{j}}{j!}{\left(b-1\right)}^{p-k}\int_{0}^{\infty }x^{k+bj-j+p\left(b-2\right)}exp\left(\left(-j-1\right)x\right)dx\right]$$

A solution to the integral form in the above expression leads to the final result

$$R_{H}\left(x\right)=\frac{1}{1-p}\log\left[{\left(a^{2}b\right)}^{p+j}\sum_{j=0}^{\infty }\sum_{k=0}^{\infty }\left(\begin{array}{c} p\\ k \end{array}\right)\frac{{\left(-1\right)}^{j}}{j!}{\left(b-1\right)}^{p-k}\right]\frac{\Gamma \left(p(b-2)+k+bj-j+1\right)}{{\left(-j-1\right)}^{\left(p(b-2)+k+bj-j+1\right)}}$$ (9.2)

## Applications

This section illustrates the usefulness of *NFE*(*a*,*b*) distribution by using two real data sets. The comparison with other distributions (Exponential, Weibull Exponential and Exponentiated Exponential distributions) have been studied by using different criteria including Akaike information criterion (AIC), Consistent Akaike Information Criterion (CAIC), Bayesian information criterion (BIC), and Hannan Quinn information criterion (HQIC). For a more detailed discussion on these criteria and their applications to various fields, we refer to see [13–20]. The mathematical form of these criteria are given by

$$AIC=-2L+2p,\;AICc=AIC+\frac{2p(p+1)}{n-p-1},\;CAIC=-2L+P\left\{\log(n)+1\right\}$$

BIC=Plog(n)−2L,HQIC=−2L+2Plog{log(n)}.

where, $L=L(\hat{\psi };y_{i})$ is the maximized likelihood function and *y*_*i*_ is the given random sample, $\hat{\psi }$ is the maximum likelihood estimator and *p* is the number of parameters in the model.

As a general rule, a probability model with fewer values of these criteria should be considered the best-fitted model among other probability distributions.

### Data set 1: Failure times of Aircraft windshield

The first data set represents the failure times of 84 Aircraft windshields recently studied by Ramos et. al [21]. The data set values are 0.040, 1.866, 2.385, 3.443, 0.301, 1.876, 2.481, 3.467, 0.309,1.899, 2.610, 3.478, 0.557, 1.911, 2.625, 3.578, 0.943, 1.912,2.632, 3.595, 1.070, 1.914, 2.646, 3.699, 1.124, 1.981, 2.661,3.779,1.248, 2.010, 2.688, 3.924, 1.281, 2.038, 2.82,3, 4.035, 1.281, 2.085, 2.890, 4.121, 1.303, 2.089, 2.902, 4.167, 1.432,2.097, 2.934, 4.240, 1.480, 2.135, 2.962, 4.255, 1.505, 2.154,2.964, 4.278, 1.506, 2.190, 3.000, 4.305, 1.568, 2.194, 3.103,4.376, 1.615, 2.223, 3.114, 4.449, 1.619, 2.224, 3.117, 4.485,1.652, 2.229, 3.166, 4.570, 1.652, 2.300, 3.344, 4.602, 1.757,2.324, 3.376, 4.663.

**Fig 3:**
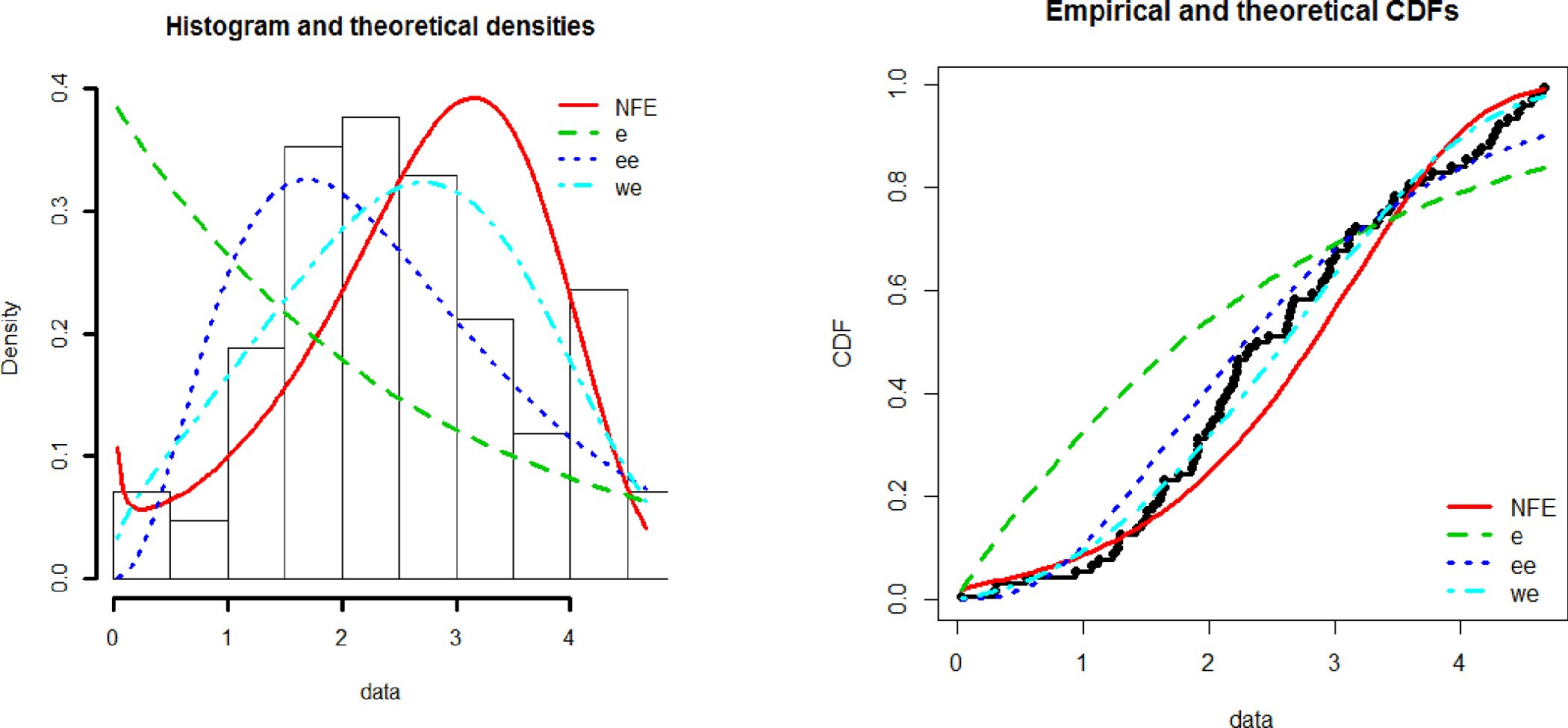
Histogram, theoretical density, empirical and theoretical CDF for NFE.

**Fig 4:**
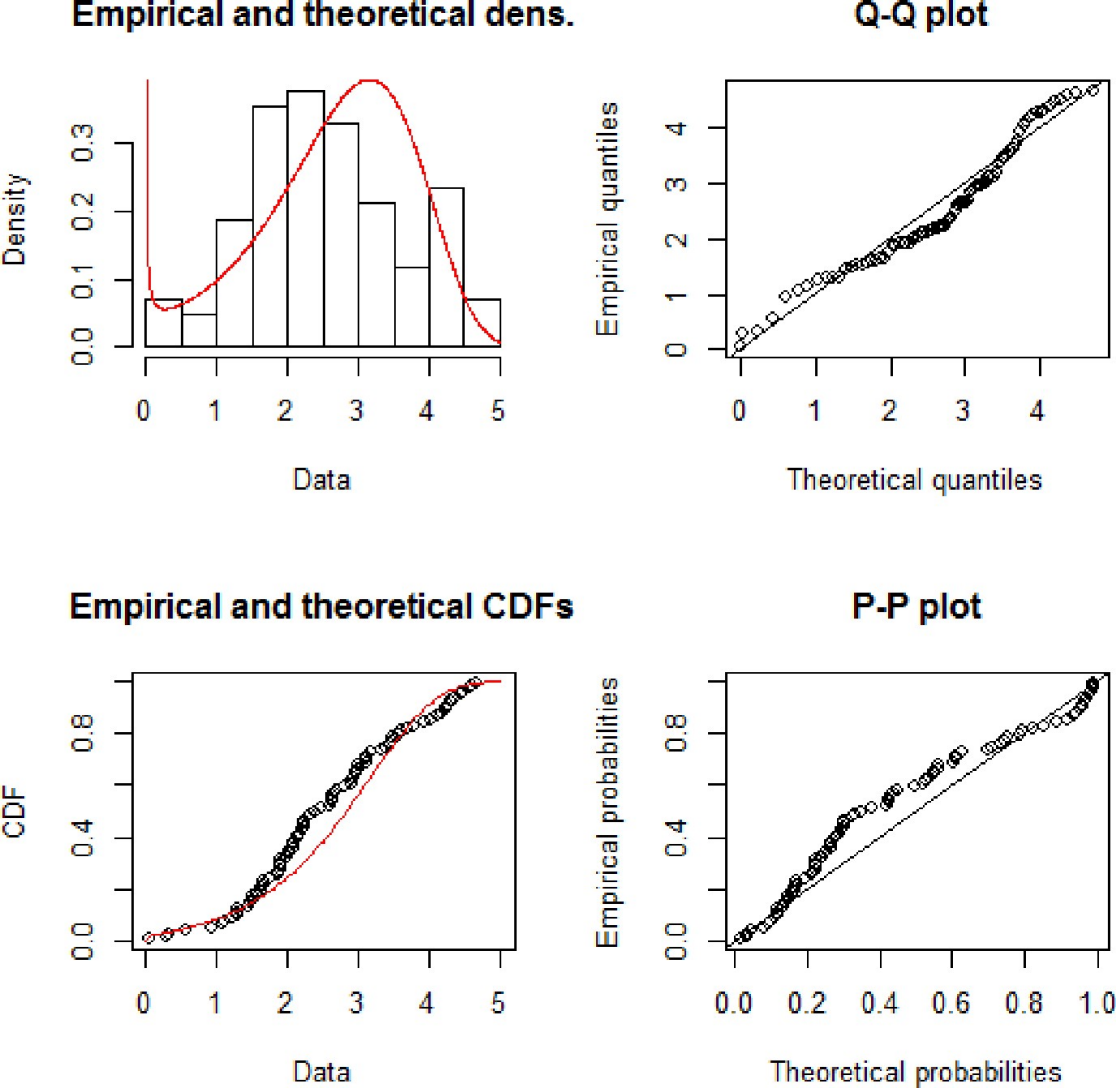
Theoretical and empirical Pdf and Cdf with Q-Q plot and P-P plot for NFE.

Fig 3 shows the empirical and theoretical Cdf and Pdf of NFE distribution while Fig 4 represents the QQ and PP plots. Table 1 represents the maximum likelihood estimates of the New Flexible Exponential distribution for aircraft data. Table 2 represents the goodness of fit criteria including AIC, CAIC, BIC, and HQIC. The numerical values in Table 2 are less for the New Flexible Exponential distribution than others and hence we conclude that the New Flexible Exponential distribution perform better as compared to Exponential, Weibull Exponential and the Exponentiated Exponential distribution.

**Table 1:** Maximum likelihood estimates for aircraft data.

Model	Estimates		
*NFE*(*a*,*b*)	-0.1652185	1.2086781	_
*EE*(*a*,*b*)	0.7579791	3.5930709	_
*E*(*a*)	0.3902274	_	_
*WE*(*a*,*b*,*c*)	0.05827534	3.40973109	0.26963313

**Table 2:** Goodness of fit criteria, AIC, CAIC, BIC, HQIC for aircraft data.

Model	*AIC*	*CAIC*	*BIC*	*HQIC*
*NFE*(*a*,*b*)	269.9814	270.1277	274.8667	271.9464
*EE*(*a*,*b*)	286.7922	286.9385	291.6775	288.7572
*E*(*a*)	331.9754	332.0236	334.418	332.9579
*WE*(*a*,*b*,*c*)	270.3205	270.6168	277.6485	273.268

### Data set 2: Strengths of 1.5 cm glass bares

The second real data set represents the Strengths of 1.5 cm glass bares, measured at the National Physical Laboratory, England. The data set is taken from the Smith and Naylor [22] with the following values 0.55,0.93,1.25,1.36,1.49,1.52,1.58,1.61,1.64,1.68,1.73,1.81,2,0.74,1.04,1.27,1.39,1.49,1.53,1.59,1.61,1.66,1.68,1.76,1.82,2.01,0.77,1.11,1.28,1.42,1.5,1.54,1.6,1.62,1.66,1.69,1.76,1.84,2.24,0.81,1.13,1.29,1.48,1.5,1.55,1.61,1.62,1.66,1.70,1.77,1.84,0.84,1.24,1.3,1.48,1.51,1.55,1.61,1.63,1.67,1.7,1.78,1.89.

**Fig 5:**
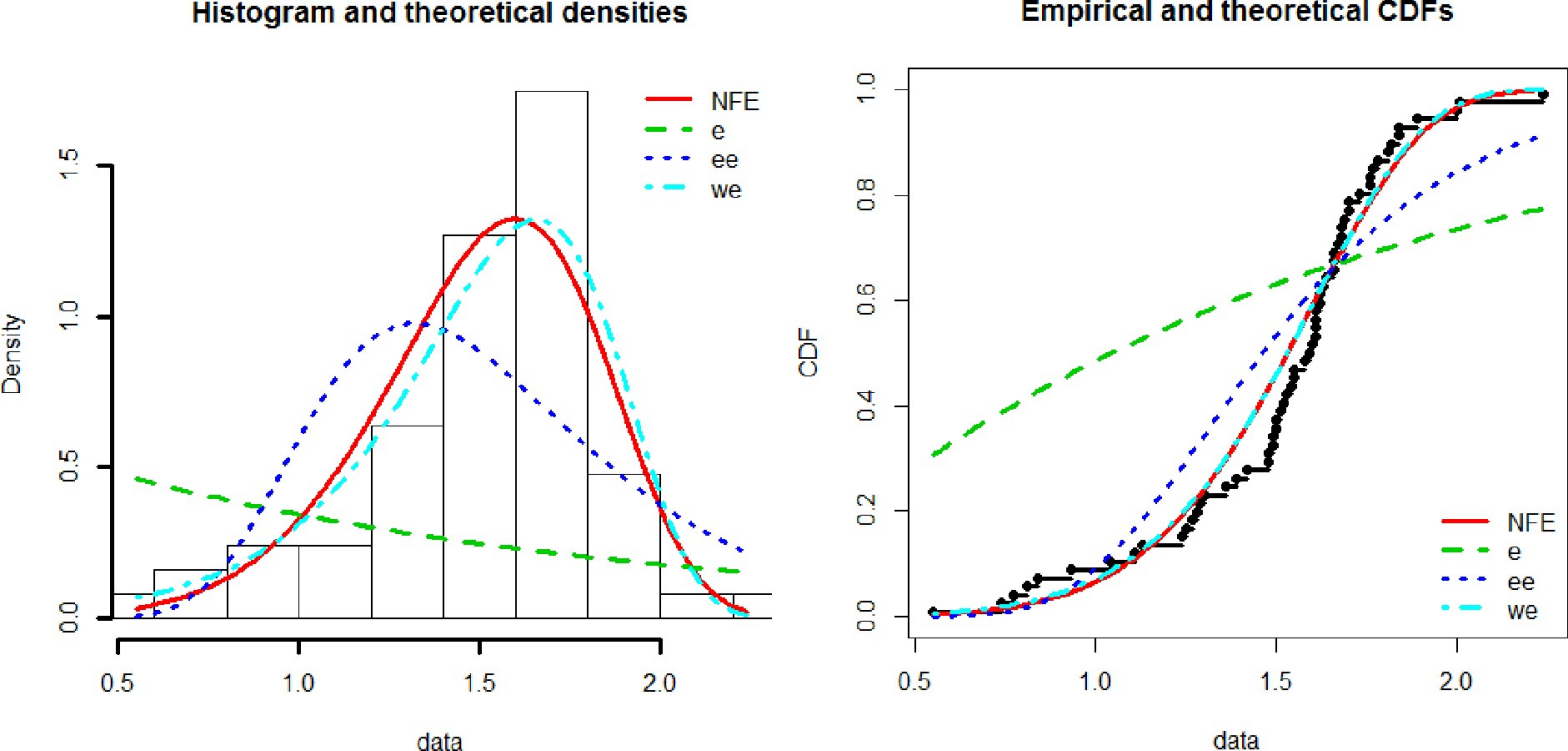
Histogram, theoretical density, empirical and theoretical CDF for NFE.

**Fig 6:**
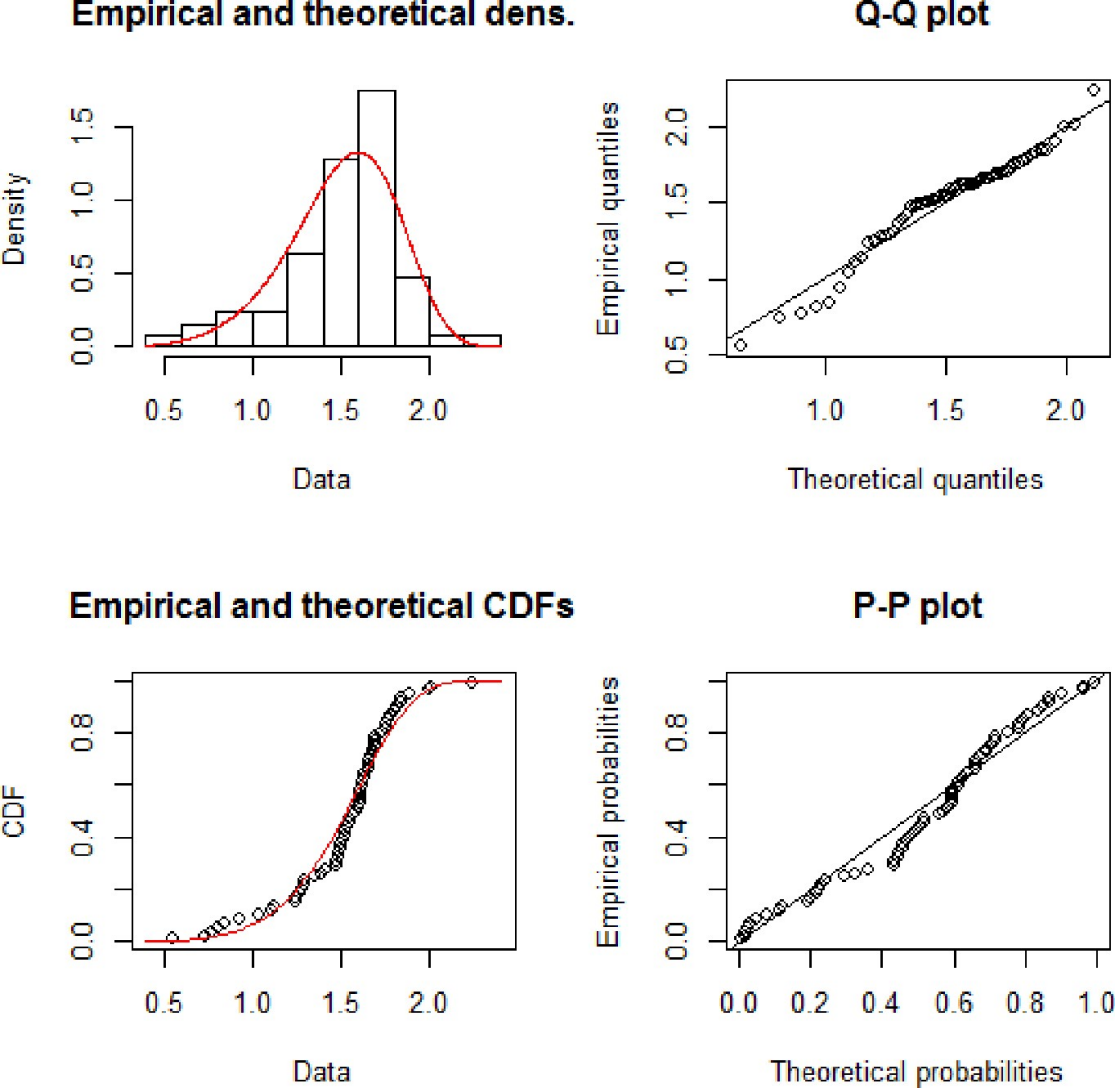
Theoretical and empirical Pdf and Cdf with Q-Q plot and P-P plot for NFE.

Fig 5 shows the empirical and theoretical Cdf and Pdf of NFE distribution while Fig 6 represents the QQ and PP plots. Table 3 represents the maximum likelihood estimates of the New Flexible Exponential distribution for aircraft data. Table 4 represents the goodness of fit criteria including AIC, CAIC, BIC, and HQIC. The numerical values in Table 4 are less for the New Flexible Exponential distribution than others and hence we conclude that the New Flexible Exponential distribution perform better as compared to Exponential, Weibull Exponential and the Exponentiated Exponential distribution.

**Table 3:** Maximum likelihood estimates for glass bares data.

Model	Estimates		
*NFE*(*a*,*b*)	-0.06832238	5.22227388	_
*EE*(*a*,*b*)	2.609189	31.235128	_
*E*(*a*)	0.6636407	_	_
*WE*(*a*,*b*,*c*)	0.02490157	0.87090869	3.15914456

**Table 4:** Goodness of fit criteria: AIC, CAIC, BIC, HQIC for glass bares data.

Model	*AIC*	*CAIC*	*BIC*	*HQIC*
*NFE*(*a*,*b*)	33.23809	33.43809	37.52436	34.9239
*EE*(*a*,*b*)	66.76709	66.96709	71.05336	68.4529
*E*(*a*)	179.6606	179.7262	181.8038	180.5035
*WE*(*a*,*b*,*c*)	35.32628	41.34891	41.34891	37.44822

## Simulation

To conduct a simulation study, Eq (4.2) is used to generate random data from the New Flexible Exponential distribution. The simulation experiment is repeated for 100 times each with sample of size *n* = 120, 150 and 180. First, we fixed the parameter *b* = 0.3 and vary *a* = 0.008,0.009,0.01,0.02 0.3. Secondly, we fixed the variable *a* = 0.01 and vary *b* = 0.4,0.44,0.5,0.51. Table 5 demonstrates the mean Bias and Mean square error (MSE). The result given in Table 5 has shown that both the Bias and MSE are decreasing as the sample size n increase.

**Table 5:** Mean Bias and MSE of *NFE*(*a*,*b*) distribution.

*a*	*b*	*n*	*MSE*(*a*)	*MSE*(*b*)	*Bias*(*a*)	*Bias*(*b*)
0.008	0.3	120	239.732	1.270981	15.33756	1.127165
		150	198.0171	1.238176	14.0047	1.112614
		180	132.6051	1.175595	11.51539	1.084249
0.009		120	239.7993	1.295611	15.46873	1.138219
		150	166.1738	1.226752	12.84548	1.107504
		180	145.187	1.221275	12.04171	1.105095
0.01		120	223.315	1.309414	14.90223	1.14421
		150	216.3098	1.307236	14.66495	1.143251
		180	158.3899	1.256698	12.56349	1.120984
0.02		120	178.2621	1.416837	13.28176	1.190151
		150	147.1883	1.411141	12.11266	1.187821
		180	139.8459	1.402829	11.81408	1.184358
0.01	0.4	120	182.2544	0.9568964	13.48662	0.9781792
		150	123.7268	0.9089113	11.12114	0.9533604
		180	119.571	0.9017053	10.88265	0.9494812
	0.44	120	134.648	0.7971483	11.59609	0.8928112
		150	121.4153	0.7899957	10.96512	0.8887261
		180	93.37407	0.7614382	9.597455	0.872489
	0.51	120	103.7858	0.5961089	10.17505	0.7720555
		150	85.96842	0.5823062	9.225429	0.7630093
		180	77.33886	0.57584	8.792402	0.7588345
	0.5	120	110.3115	0.623783	10.48543	0.7897792
		150	84.10164	0.602161	9.14759	0.7759354
		180	80.59911	0.6047069	8.976754	0.7776268

## Conclusion

In this paper, a new family of distribution called Exponential-H (Ex-H) family of distribution is presented. The special case is derived by employing the Weibull distribution as a baseline distribution and we called it New Flexible Exponential distribution (NFE). Different statistical properties of the NFE distribution are obtained such as hazard function, Survival function, order statistics, moments, and Renyi entropy. The parameters of the model are estimated using the maximum likelihood method. Moreover, the simulation study is also carried out. Two data sets were used to support the usefulness of the NFE distribution. The numerical values conclude that the NFE distribution performed better than Exponential, Weibull Exponential, and Exponentiated Exponential distribution.

## Supporting information

S1 Data Failure times of Aircraft windshield [21]. (TIF)

S2 Data Strengths of 1.5 cm glass bares [22]. (TIF)
