# Supplementary figures and images for: A novel family of lifetime distribution with applications to real and simulated data
Source: PLoS One. 2020 Oct 1;15(10):e0238746. doi: 10.1371/journal.pone.0238746 (PMC7529267; doi:10.1371/journal.pone.0238746)

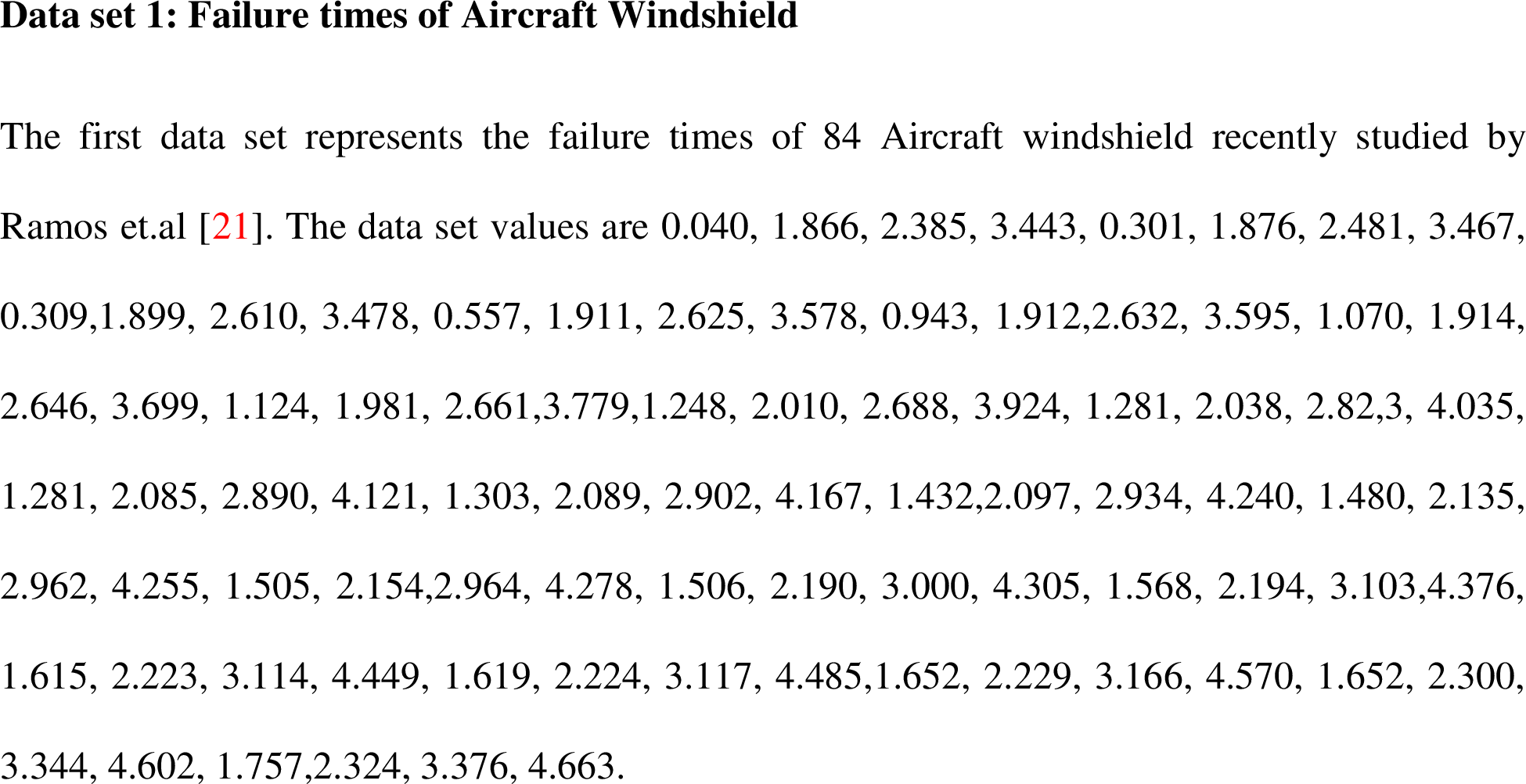

Supplement: S1 Data — (TIF) [file pone.0238746.s001.tif]

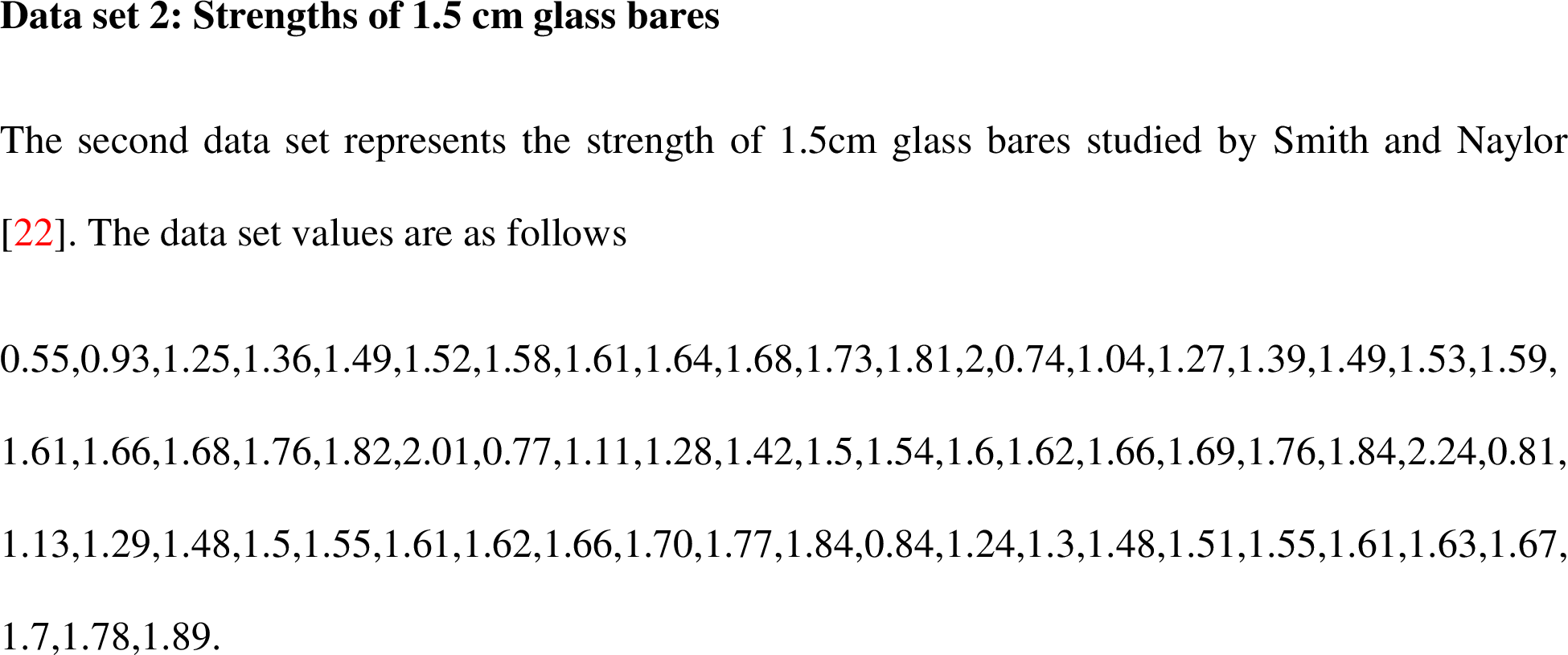

Supplement: S2 Data — (TIF) [file pone.0238746.s002.tif]
